# “Uroptysis!” – A case report of xanthogranulomatous pyelonephritis with nephrobronchial fistulation

**DOI:** 10.1016/j.ijscr.2022.107551

**Published:** 2022-08-26

**Authors:** S. O'Neill, R. Motyer, H. O'Neill, I. Brennan, J.M. Ryan, M. Guiney

**Affiliations:** Department of Interventional Radiology, St. James's Hospital, Dublin, Ireland

**Keywords:** Case report, Xanthogranulomatous pyelonephritis, Nephrostomy, Nephrobronchial fistula

## Abstract

**Introduction and importance:**

Xanthogranulomatous pyelonephritis (XGP) is an uncommon complication of chronic urinary tract infection, classically secondary to a chronic obstructive uropathy, resulting in destruction of renal parenchyma and a non-functioning kidney (Jha and Aeddula, 2022 [1]). This is rarely associated with nephrobronchial fistulation, with few published case reports in the literature to date.

**Case presentation:**

We present the rare case of a 42-year-old female who was admitted to an Irish tertiary urology centre with abdominal pain, elevated inflammatory markers and an obstructive uropathy on initial imaging, with a new diagnosis of XGP. Initial management was with targeted intravenous antimicrobial therapy, percutaneous nephrostomy and perinephric drain insertion. The subsequent discovery of a nephrobronchial fistula later complicated the case, warranting plan for interval nephrectomy and fistula repair following prolonged medical management. We discuss the initial presentation, workup and image-guided approach to management.

**Clinical discussion:**

XGP is an uncommon sequela of chronic renal suppurative infection, and is usually associated with long-standing ureteric obstruction secondary to a staghorn calculus. Nephrobronchial fistulation is a rare complication of XGP, highlighting the significance of this case discussion.

**Conclusion:**

XGP should be considered in cases of suspected chronic pyelonephritis and may rarely lead to nephrobronchial fistulation. In cases of known XGP and pleural empyema, nephrobronchial fistulation should be considered as part of the differential diagnosis.

## Highlights

• Xanthogranulomatous pyelonephritis is rare and associated with chronic upper urinary tract infection and obstruction.
• Nephrobronchial fistulation is a rare complication of xanthogranulomatous pyelonephritis.
• In cases of XGP and concurrent pleural empyema, a nephrobronchial fistula should be considered.

## 1 Introduction

XGP is a rare complication of chronic pyelonephritis resulting in destruction of the renal parenchyma. It is most commonly associated with a chronic obstructive uropathy secondary to a staghorn calculus, and *Proteus Mirabilis* or *Escherichia Coli* urinary tract infection [1], [2]. Parenchymal destruction can be so severe that it can lead to fistula formation and invasion into surrounding structures, with nephrobronchial fistulation being a very rare consequence of XGP [3]. Standard management is with targeted antimicrobials, abscess drainage and surgical intervention with nephrectomy and fistula repair in severe cases [3]. This case highlights the significant complications which can follow untreated urinary tract infection and nephrolithiasis, and demonstrates that XGP should be considered in all patients with chronic or complicated pyelonephritis.

## 2 Patient information

A 42-year-old Caucasian female self-presented to the emergency department of an Irish tertiary urology centre with a three-week history of abdominal pain, nausea and vomiting. This was associated with posterior thoracic pain, a productive cough and shortness of breath over the same period. Family history was non-contributory. Regular medications included diazepam 2.5 mg once daily, pregabalin 25 mg three times daily and citalopram 20 mg once daily. Medical history included gestational diabetes mellitus, while surgical history included an uncomplicated elective open reduction and internal fixation for an explained fibular fracture. The patient was living independently in an urban area, regularly smoked tobacco, did not consume alcohol and was not in employment at this time.

## 3 Clinical findings

Clinical examination on admission revealed a soft abdomen with generalised tenderness and no palpable masses or evidence of peritonitis. Bowel sounds were present and unremarkable. Auscultation of the posterior thorax revealed reduced air entry in the left base with coarse crackles to the left mid zone. Percussion note was dull over the left base with reduced vocal resonance. No other pertinent signs were noted on examination.

## 4 Timeline

The patient described the abdominal pain as beginning three weeks prior to presentation, being constant in nature and worsening gradually during this time. She did not seek medical assistance prior to this presentation.

## 5 Differential diagnosis

Initial differential diagnosis included parapneumonic or malignant pleural effusion. Chest radiograph and cross sectional imaging of the abdomen and pelvis were requested to further evaluate this.

## 6 Diagnostic assessment

**Fig. 1:**
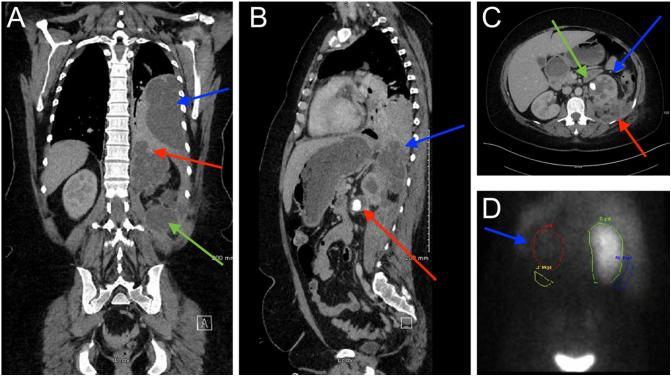
A: Coronal contrast enhanced CT demonstrating large loculated left-sided pleural effusion with extensive compressive atelectasis (blue arrow), dilated left renal calyces and perirenal collections extending superiorly through defect in the left hemidiaphragm (red arrow), and multiple pararenal organised collections (green arrow). B: Sagittal images from same CT demonstrating perirenal collections extending through the left hemidiaphragm defect to the left hemithorax (blue arrow), obstructing PUJ calculus (red arrow). C: Axial images from same CT demonstrating oedematous poorly enhancing left kidney with calyceal dilation giving multiloculated appearance (bear paw sign), consistent with XGP (blue arrow). Obstructing calculus at the left PUJ (green arrow). Multiloculated left-sided perirenal and pararenal collections (red arrow). D: DMSA image demonstrating no uptake in the left kidney (blue arrow), consistent with diagnosis of XGP. (For interpretation of the references to colour in this figure legend, the reader is referred to the web version of this article.)

Initial laboratory investigations were significant for elevated serum inflammatory markers and elevated urea and creatinine consistent with an acute kidney injury. There was no growth on serial mid-stream urine cultures. Chest radiograph demonstrated an extensive left-sided pleural effusion. CT abdomen and pelvis on admission showed an obstructing renal calculus in the left pelviureteric junction with associated peri-splenic and pararenal abscess formation, and a left-sided pleural effusion with compressive atelectasis (Fig. 1). DMSA imaging showed a non-functioning left kidney and a diagnosis of XGP was made (Fig. 1).

## 7 Therapeutic intervention

Initial management involved intravenous piperacillin-tazobactam 4.5 g twice daily, with ultrasound-guided left-sided percutaneous perinephric drain and nephrostomy insertion by a senior interventional radiology trainee. The purposes of these procedures were for infectious source control and relief of ureteric obstruction respectively. Nephrostogram at the time of nephrostomy insertion demonstrated interpolar calyceal rupture. Post-procedure instructions included flushing the nephrostomy three times daily with 10 ml 0.9 % sodium chloride, and monitoring of nephrostomy and drain output. Culture of drain fluid later grew *Proteus mirabilis* susceptible to amoxicillin-clavulanic acid, however the patient was intolerant of this antimicrobial. Regular analgesia was also used for pain control, with intravenous paracetamol 1 g four times daily and oral oxycodone 2.5 mg as required. The interventions were well tolerated by the patient and pain was well controlled.

The patient remained clinically unwell during her stay, with persistently elevated inflammatory markers, warranting repeat imaging. CT TAP demonstrated an evolving left-sided empyema which was subsequently drained percutaneously. Respiratory arrest occurred at the time of percutaneous chest drain insertion and the patient was successfully resuscitated, requiring a brief ICU admission. The exact cause of the respiratory arrest was not immediately identified. Culture of drain fluid revealed no growth, however 16S rRNA analysis later identified *Proteus* species.

**Fig. 2:**
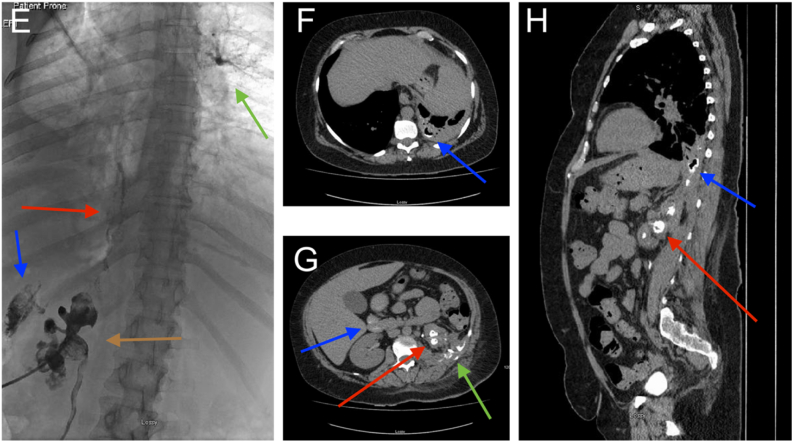
E: Fluoroscopic image from left nephrostogram (patient prone) demonstrating contrast filling left collecting system, with filling defects likely representing blood clots and debris (brown arrow), extrarenal contrast secondary to calyceal rupture (blue arrow), extrarenal contrast extending from upper pole to left hemidiaphragm (red arrow), contrast within the right bronchial tree, consistent with nephron-bronchial fistula (green arrow). The patient was noted to cough at time of nephrostogram F: Axial image from non-contrast enhanced CT immediately following nephrostogram demonstrating contrast pooling in cavitation within the atelectatic left lower lobe (blue arrow). G: Axial image from same non-contrast enhanced CT demonstrating contrast in the left renal calyces (red arrow), pararenal contrast secondary to ruptured calyces (green arrow), intraluminal contrast in the proximal duodenum secondary to aspiration (blue arrow). H: Axial image from same non-contrast enhanced CT demonstrating contrast in the left renal calyces (red arrow), contrast extending through defect in the left hemidiaphragm to left lower lobe cavitation and peripheral segmental bronchi (blue arrow). (For interpretation of the references to colour in this figure legend, the reader is referred to the web version of this article.)

The patient clinically improved and antegrade ureteric stenting was planned to facilitate nephrostomy removal, with view to ongoing conservative management and discharge. Nephrectomy was not performed due to the severity of the perinephric inflammation. A nephrostogram was performed prior to ureteric stent insertion which demonstrated persistent calyceal rupture, with contrast tracking from the upper pole of the left kidney across the left hemidiaphragm and opacifying the right bronchial tree (Fig. 2). The patient began coughing during the procedure. Immediate post-procedure CT TAP confirmed a left nephrobronchial fistula (Fig. 2).

## 8 Follow up and outcomes

The nephrostomy was left in situ, and the patient was later discharged when clinically and biochemically stable with outpatient intravenous ceftriaxone 2 g once daily. Outpatient urology clinical follow up was scheduled for one-week post-discharge and a plan made for interval nephrectomy and fistula repair. The patient was also followed in the infectious diseases clinic ten days post-discharge as part of the national outpatient antimicrobial therapy programme, with regular clinical review and monitoring of serum inflammatory markers, which down-trended. There was no follow-up cross-sectional imaging completed at the time of writing this report. There were no significant complications or adverse events noted post-discharge.

## 9 Discussion

XGP is a rare complication of chronic urinary tract infection and obstruction, the incidence of which has significantly reduced since the widespread use of antibiotics [1]. Approximately 90 % of urine cultures from patients presenting with the disease will grow *Proteus mirabilis* or *Escherichia coli*, however many patients have no growth on urine culture, as is seen in this case [2]. It is more common in women than men, and most commonly presents with a prolonged history of abdominal or flank pain, often on a background of recurrent urinary tract infections. The lack of clear clinical history of recurrent urinary tract infections or nephrolithiasis in this case demonstrates an insidious presentation of the disease. Computer tomography imaging is the diagnostic imaging modality of choice, where the radiological appearance of the disease, as well as its insidious presentation, can mimic malignancy in many cases [4], [5]. Treatment typically involves prolonged courses of antimicrobials, with nephrectomy being the definitive treatment modality to avoid infectious complications such as abscess formation [6].

Nephrobronchial fistula formation is a rare complication of XGP, with a limited number of published case reports to date. It should however be considered in any case of known XGP with coexisting pleural empyema or an inappropriately slow response to treatment. Nephrobronchial fistulas are best visualised through imaging modalities where they can be directly opacified with contrast, such as through contrast injection during nephrostogram as is seen in this case. Interestingly, we postulate that the respiratory arrest at time of chest drain insertion may have been secondary to the bronchial fistulation, with possible mucus plugging or debris causing airway occlusion at time of intervention at this site.

## 10 Learning points

XGP is a rare consequence of chronic urinary tract infection and obstruction and should be considered in cases of chronic or complicated pyelonephritis. XGP may lead to aggressive local inflammation with resulting complications, such as nephrobronchial fistulation, as in this case. Optimal diagnosis of a nephrobronchial fistula is with nephrostogram if a nephrostomy is in situ, however close attention on cross-sectional imaging may demonstrate fistulous tracts. Nephrobronchial fistula formation should be considered in all patients with known XGP and concurrent pleural empyema.

Our case report highlights this rare clinical entity and raises awareness to the severity of potential complications of XGP. We would be interested in further research on this topic at a national level, particularly regarding XGP outcomes and optimal treatment paradigms.

## 11 Patient perspective

The patient was continuously reassured throughout their prolonged hospital admission and had total insight into the severity and rarity of their disease. They were most affected emotionally by the experience of ICU admission and the severity of their illness during this time. However, they remained in good spirits throughout their hospital stay and looked forward to further engagement with the relevant outpatient services on discharge.

## 12 Additional information

This work has been reported in line with the SCARE 2020 criteria [7].

## Provenance and peer review

Not commissioned, externally peer reviewed.

## Funding

No sponsorship was required for this study.

## Ethical approval

This study was exempt from ethical approval under St James's Hospital/Tallaght University Hospital Joint Research Ethics Committee policy as a retrospective case report.

## Consent

Written informed consent was obtained from the patient for publication of this case report and accompanying images. A copy of the written consent is available for review by the Editor-in-Chief of this journal on request.

## Author contribution

**Susan O'Neill**: writing – original draft; writing – review and editing.

**Ronan Motyer**: writing – review and editing; project administration.

**Hazel O'Neill**: image acquisition and formatting.

**Ian Brennan**: supervision and edits.

**Mark Ryan**: supervision and edits.

**Michael Guiney**: supervision and edits.

## Registration of research studies

N/A.

## Guarantor

Susan O'Neill, Ronan Motyer.

## Declaration of competing interest

The authors had no conflicts of interest to declare.
